# Lung adenocarcinoma with brain metastasis detected dual fusion of LOC399815-ALK and ALK-EML4 in combined treatment of Alectinib and CyberKnife: A case report

**DOI:** 10.1097/MD.0000000000036992

**Published:** 2024-01-19

**Authors:** Yumei Li, Shijin Lu, Ping Yao, Wenchuang Huang, Yong Huang, Ying Zhou, Ying Yuan, Shaochen Cheng, Fasheng Wu

**Affiliations:** a Ruikang Hospital Affiliated to Guangxi University of Traditional Chinese Medicine, Nanning, China; b Guangxi Medical University Cancer Hospital, Nanning, China; c HaploX Biotechnology, Shenzhen, China.

**Keywords:** Alectinib, ALK fusion, ctDNA, LOC399815-ALK, nonsmall cell lung cancer, stereotactic radiosurgery

## Abstract

**Introduction:**

The anaplastic lymphoma kinase (ALK) gene fusion occurs in approximately 3% to 7% of nonsmall cell lung cancer (NSCLC), in which occurs approximately 23% to 31% of brain metastasis patients in poor prognosis. ALK tyrosine kinase inhibitors have shown efficacy in treating ALK-positive (ALK+) NSCLC. More than 90 distinct subtypes of ALK fusions have been identified through sequencing technique and would lead to significant differences in clinical efficacy, it is necessary to guide clinical treatment effectively by gene detection.

**Patient concerns:**

A 56-year-old nonsmoking female admitted to hospital due to cough, expectoration, and chest pain. Chest computed tomography revealed a space-occupying lesion in the upper left lobe (5.0 cm × 2.4 cm × 2.9 cm), multiple enlarged lymph nodes in mediastinum 3A and 5 (largest size 1.5 cm × 1.4 cm), and evidence of thoracic vertebral metastasis, brain magnetic resonance imaging also showed brain metastasis.

**Diagnoses:**

Lung adenocarcinoma with brain metastasis.

**Interventions:**

The patient initially received conventional first-line chemotherapy, which led to a deteriorated condition. Blood-base liquid biopsy by next-generation sequencing resulted in double ALK fusions, in which with a neo-partner of lncRNA (LOC399815-ALK). Following subsequent treatment with Alectinib and stereotactic radiotherapy (CyberKnife) was subsequently employed to manage the brain metastatic lesions, resulting in a substantial decreased in both the number and size of tumor lesions.

**Outcomes:**

The patient’s response to therapy efficacy resulted in a substantial decreased in both the number and size of tumor lesions that assessed comprehensively evaluated through computed tomography imaging and ctDNA sequencing. Patient’s condition has been under control for over 29 months.

**Conclusion:**

Liquid biopsy may reveal the rare fusion forms of ALK, precisely guiding personalized treatment, and providing a reference method for longitudinal monitoring and efficacy evaluation of ALK-tyrosine kinase inhibitors in NSCLC patients.

## 1. Introduction

Nonsmall cell lung cancer (NSCLC) constitutes approximately 80% of all lung cancer, with anaplastic lymphoma kinase (ALK) fusion gene-positive NSCLC accounts for roughly 3% to 7% of cases.^[1]^ Next-generation sequencing (NGS) has identified over 90 ALK-fusion subtypes, with the majority being single variants. Concurrent presence of 2 or more ALK-fusion forms is rare.^[2,3]^ Echinoderm microtubule-associated protein 4 (EML4) is the most frequently fusion partner of ALK fusion.^[4]^ Additionally, brain metastasis occurs in approximately 23% to 31% of patients with ALK-fusion NSCLC and is associated with a poor prognosis.^[5,6]^ ALK tyrosine kinase inhibitors (TKIs) exhibit therapeutic effects in patients with ALK fusion and potentially leading to significant improvements in the survival and prognosis of those with ALK-positive (ALK+) NSCLC.

The second-generation ALK-TKIs, Alectinib, has been shown a good therapeutic effect on intracranial lesions because of its advantage of penetrating the blood-brain barrier.^[7]^ While NSCLC is characterized as high heterogeneity and the sensitivity of ALK-TKIs in patients with different types of ALK-fusion variant subtypes leading to significant differences in clinical efficacy.^[3]^ Therefore, it is necessary to guide clinical treatment effectively by gene detection.

Here, we report a 56-year-old lung adenocarcinoma female patient with brain metastasis and dual ALK fusion of LOC399815-ALK and ALK-EML4. Initial attempts utilizing standard chemotherapy has worsen to patient’s condition. However, subsequent administration of Alectinib significantly decreased the number and size of the main lung lesions. After stereotactic radiosurgery (CyberKnife) was combined to treat brain metastases, satisfactory therapeutic results were obtained.

## 2. Case presentation

**Figure 1.**
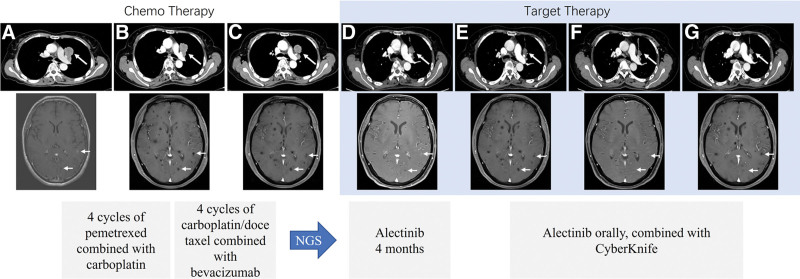
Dynamic chest CT scan (up), brain MRI imaging (down) and treatment timeline of patients during treatment. Chest CT and brain MRI at initial diagnosis (A). After 4 cycles of pemetrexed combined with carboplatin treatment, the size of left upper lung lesions decreased, but the number of brain lesions increased (B). After 4 cycles of carboplatin/docetaxel combined with bevacizumab treatment, the size of the left upper lung lesion was stable, but the brain lesion continued to deteriorate (C). Four months target therapy of treatment with Alectinib, the size of the left upper lung lesions was reduced, the brain lesions was significantly reduced in number and size (D). After 8 months of treatment with Alectinib, the left upper lung lesions remained unchanged. However, the number and size of brain lesions increased again (E). Take Alectinib orally combined with CyberKnife for 7 months, the left upper lung lesion was continually shrunk, the number and size of brain lesions were reduced (F). After 29 months of treatment with Alectinib combined with CyberKnife, the left upper lung lesions stayed stable, number of brain lesions increased slightly, but no local enhancement was found on enhanced MRI (G). CT = computed tomography, MRI = magnetic resonance imaging.

**Figure 2.**
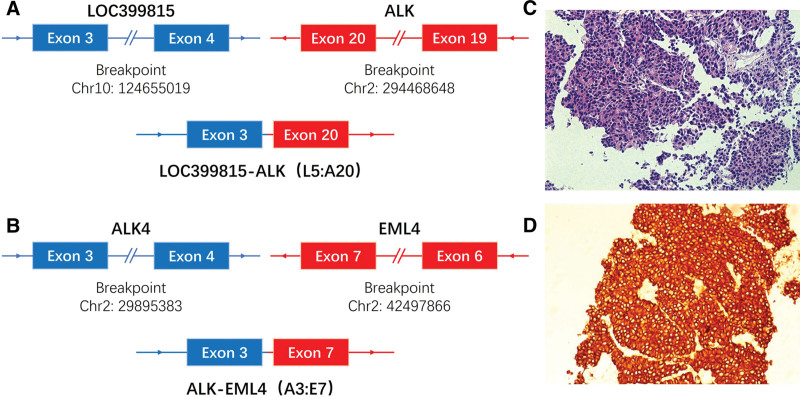
NGS and immunohistochemical findings of the lung-tumor-tissue samples. (A) ALK gene and the LOC399815 gene map to chromosome 2 and 10, break point of LOC399815 at chr10 124,655,019 and is ligated to a position of chr2 294,468,648 of ALK, giving rise to the LOC399815–ALK fusion. (B) ALK gene and the EML4 gene map to chromosome 2, break point of EML4 at chr2 42,497,866 and is ligated to chr2 29,895,383 of ALK, giving rise to the ALK-EML4 fusion. (C) Hematoxylin and eosin stain of lung biopsy. (D) Immunohistochemistry (IHC) stain with anti-ALK antibody of lung biopsy. ALK = anaplastic lymphoma kinase, NGS = Next-generation sequencing.

In June 2020, a 56-year-old female patient with no history of smoking history and familiar cancer was hospitalized due to “cough, expectoration and chest pain.” Chest computed tomography (CT) revealed a space-occupying lesion in the upper left lobe (5.0 cm × 2.4 cm × 2.9 cm), multiple enlarged lymph nodes in mediastinum 3A and 5 (largest size 1.5 cm × 1.4 cm), and evidence of thoracic vertebral metastasis (Fig. 1A). Additionally, brain magnetic resonance imaging (MRI) showed brain metastasis (Fig. 1A). Under CT-guided percutaneous fine-needle lung biopsy, the pathological results tended to be lung adenocarcinoma, with ≥200 cancer cells (Fig. 2C). Immunohistochemical staining showed ALK positive (ALK monoclonal antibody D5F3, Ventana) (Fig. 2D). The patient was ultimately diagnosed with IVB lung adenocarcinoma (cT2bN2M1c). Four cycles (D1/3W) of chemotherapy were performed from June 23 to September 1, 2020: Pemetrexed (500 mg/m^2^) combined with Carboplatin (area under the plasma concentration time curve [AUC], 5 mg/mL/min). On September 23, 2020, CT scan showed the progression of brain tumors (Fig. 1B). Next, the patient was received 4 cycles (D1/3W) of docetaxel (75 mg/m^2^) combined with carboplatin (AUC, 5 mg/mL/min) and bevacizumab (15 mg/kg).

On January 2021, CT scan demonstrated stable control of the thoracic lesions (Fig. 1C), yet there was an escalation in the severity of intracranial lesions (Fig. 1C). The ctDNA in peripheral blood was conducted by NGS (680-gene target-capturing panel, coverage 2200×), unveiling double ALK rearrangements of LOC399815-ALK (L5: A20, VAF 8.16%) and ALK-EML4 (A3: E7, VAF 2.51%) (Fig. 2A,B; Table 1). Additional SNV (HIF1A, NOTCH1, GEN1, PDCD1, and RBM10) and CNV mutations of deletion (CDKN2A, CDKN2B, CHEK2, CRKL, and NF2) and amplification (AR, ATRX) were also identified (Table 1). According to NGS results, patient received 4 months targeted treatment with second-generation ALK-TKIs of Alectinib from February 2021 (600 mg twice a day). The primary tumor decreased to 2.1 cm × 1.3 cm × 1.7 cm by chest CT on June 24 (Fig. 1D). According to the evaluation criteria of solid tumor response (RECIST 1.1), the curative effect was evaluated as PR (Fig. 1D). In September 2021, brain MRI showed increased number and volume of intracranial metastatic lesions (Fig. 1E), while chest CT showed no significant change (Fig. 1E). The curative effect was determined as disease progression (PD).

**Table 1:** List of blood NGS changes before and after treatment with Alectinib.

Gene	Variation	Variation rate (%)/copy number	
		January 21, 2021	July 13, 2022
HIF1A	p.T544R	30.07%	–
NOTCH1	p.P1862L	19.59%	–
GEN1	p.E804K	1.40%	–
PDCD1	p.G176D	0.85%	–
RBM10	p.Q267Rfs*41	37.96%	–
AR	Amplification	3.4 Copy	–
ATRX	Amplification	3.4 Copy	–
CDKN2A	Deletion	0.5 Copy	–
CDKN2B	Deletion	0.5 Copy	–
CHEK2	Deletion	1 Copy	–
CRKL	Deletion	1 Copy	–
NF2	Deletion	1 Copy	–
ALK	LOC399815-exon5-ALK-exon20 fusion	8.16%	–
ALK	ALK-exon3-EML4-exon7 fusion	2.51%	–

From September 26–30 to September 2021, brain lesions were addressed with stereotactic radiosurgery (CyberKnife—24Gy/3f), while the patient concurrently maintaining an oral regimen of Alectinib (600 mg twice a day). In May 2022, MRI reexamination showed that the number and volume of brain lesions decreased (Fig. 1F), but no significant changes in lung and thoracic vertebral lesions were observed (Fig. 1F). In July 2022, the blood ctDNA NGS analysis revealed that the absence of LOC399815-ALK, ALK-EML fusion variants, as well as other concomitant mutations, with no other mutated genes being detected (Table 1). By October 2022, the brain MRI indicated an increase in the number of brain lesions (Fig. 1G), but no local lesions enhancement was found on contrast-enhanced MRI, suggesting that the lesions were inactive, and thus to continue taking Alectinib.

## 3. Discussion

Lung cancer is a highly heterogeneous tumor type. Certain rare mutations in NSCLC, such as different subtypes of ALK fusion, having a prone for treatment resistance, leading to higher recurrence rate and poor prognosis.^[8,9]^ NGS possessed ability to detect and identify rare mutation variants through high-throughput and deep sequencing.^[10]^

In the presented case, the ALK double fusion of LOC399815–ALK (L5: A20) of ALK-EML4 (A3: E7) in peripheral blood ctDNA of a lung cancer patient with brain metastasis was detected by NGS. Unlike the regular partner in ALK fusions, LOC399815 is classified as an lncRNA with 2112nt bases of 7 exons located on human chromosome 10q26.13. Previous study has reported that the expression of LOC399815 is significantly related to the clinical characteristics of patients with lung squamous cell carcinoma, including factors such as gender and lymph node metastasis.^[11]^ This fusion mode comprised exon 1–5 of lncRNA LOC399815 and exon 20–29 of ALK, the entire ALK kinase domain is preserved, like the classical EML4-ALK (E4: A20) fusion structure. It is hypothesized that the lncRNA-DNA ALK fusion may still possess activate ALK kinase, leading to the polymerization and self-phosphorylation of ALK kinase, thereby enhancing the carcinogenic potential of ALK.^[12]^ To the best of our knowledge, the lncRNA involved in ALK-fusion variant found is the first case reported. Nevertheless, lncRNA-ALK fusion is extremely rare in NSCLC, and its sensitivity and reaction duration to ALK-TKIs are unknown. While the precise molecular diagnosis by NGS and ALK-TKIs therapy have allowed the lesions controlled. In this case, the patient worsened following chemotherapy, but under Alectinib treatment the lung lesions continued to shrink, and the thoracic vertebral metastatic lesions were controlled for 29 months to date, and peripheral blood ctDNA detection revealed that the previous double fusion and other concomitant mutations to be negative.

Moreover, the brain metastatic lesions (>20) were also effectively controlled for 7 months. This suggests that Alectinib may prove effective in treating the NSCLC LOC399815-ALK variant subtype. Additionally, the combination treatment with advanced radiotherapy of CyberKnife would effectively manage the brain metastasis. Previous studies support that stereotactic radiosurgery alone may be used to treat >10 brain metastases without affecting the decline of neurocognitive function.^[13]^ With the patient receiving a combination of Alectinib and CyberKnife, a reduction of in both the volume and number of brain metastatic lesions, accompanied with an improvement in neurocognitive function. Therefore, liquid biopsy-based gene detection successfully determines rare gene fusion in clinical assistant diagnoses, such as LOC399815-ALK fusion, which guide efficient and accurate treatment and thereby improving the quality of life for NSCLC patients.^[14]^

To our notice, another ALK-EML4 (A3: E7) fusion was also identified in the blood ctDNA of this patient (Fig. 1B). This rearrangement is formed by the fusion of the third exon of ALK gene with the seventh exon of EML4 gene. Due to the lack of ALK kinase domain (exon 20–29), this fusion form may not affect the expression, transcription, and translation of ALK, and may not impact the activation of ALK. We hypothesize that ALK-EML4 (A3: E7) fusion may not response to the treatment effect of ALK-TKI, and its role in disease treatment requires further study.

## 4. Conclusion

The fusion of ALK and LOC399815 has not been reported in any tumor type. Through NGS detection, we identified a double fusion LOC399815-ALK fusion (L5: A20) and another ALK-EML4 (A3: E7) in the blood ctDNA of an NSCLC patient. The presence of the LOC399815-ALK fusion in the patient may enhance the sensitivity of Alectinib treatment. Combined with stereotactic radiosurgery (CyberKnife), Alectinib appears to be effectively control the brain metastasis in NSCLC patients with ALK fusions. The therapeutic strategies employed for this patient provide new insights for treating similar cases. Furthermore, this case underscores that liquid biopsy may reveal the rare fusion forms of ALK, guiding personalized treatment, and providing a reference method for longitudinal monitoring and efficacy evaluation of ALK-TKIs.

## Author contributions

**Writing – original draft:** Yumei Li, shijin Lu, Ping Yao, Wenchuang Huang, Yong Huang, Ying Yuan, Ying Zhou.

**Writing – review & editing:** Shaochen Cheng, Fasheng Wu.
